# Behavioral evidence of olfactory imprinting during embryonic and larval stages in lake sturgeon

**DOI:** 10.1093/conphys/coad045

**Published:** 2023-07-03

**Authors:** Jacob G Kimmel, Tyler J Buchinger, Douglas L Larson, Edward A Baker, Troy G Zorn, Kim T Scribner, Weiming Li

**Affiliations:** Department of Fisheries and Wildlife, Michigan State University, 480 Wilson Road, East Lansing MI 48824, USA; Department of Fisheries and Wildlife, Michigan State University, 480 Wilson Road, East Lansing MI 48824, USA; Department of Fisheries and Wildlife, Michigan State University, 480 Wilson Road, East Lansing MI 48824, USA; Michigan Department of Natural Resources, Marquette Fisheries Research Station, 484 Cherry Creek Road, Marquette, Michigan, 49855, USA; Michigan Department of Natural Resources, Marquette Fisheries Research Station, 484 Cherry Creek Road, Marquette, Michigan, 49855, USA; Department of Fisheries and Wildlife, Michigan State University, 480 Wilson Road, East Lansing MI 48824, USA; Department of Integrative Biology, Michigan State University, 288 Farm Lane, East Lansing MI 48824, USA; Department of Fisheries and Wildlife, Michigan State University, 480 Wilson Road, East Lansing MI 48824, USA

**Keywords:** odorants, lake sturgeon, imprinting, behavioral response

## Abstract

Many migratory fishes are thought to navigate to natal streams using olfactory cues learned during early life stages. However, direct evidence for early-life olfactory imprinting is largely limited to Pacific salmon, and other species suspected to imprint show life history traits and reproductive strategies that raise uncertainty about the generality of the salmonid-based conceptual model of olfactory imprinting in fishes. Here, we studied early-life olfactory imprinting in lake sturgeon (*Acipenser fulvescens*), which have a life cycle notably different from Pacific salmon, but are nonetheless hypothesized to home via similar mechanisms. We tested one critical prediction of the hypothesis that early-life olfactory imprinting guides natal homing in lake sturgeon: that exposure to odorants during early-life stages results in increased activity when exposed to those odorants later in life. Lake sturgeon were exposed to artificial odorants (phenethyl alcohol and morpholine) during specific developmental windows and durations (limited to the egg, free-embryo, exogenous feeding larvae and juvenile stages), and later tested as juveniles for behavioral responses to the odorants that were demonstrative of olfactory memory. Experiments revealed that lake sturgeon reared in stream water mixed with artificial odorants for as little as 7 days responded to the odorants in behavioral assays over 50 days after the initial exposure, specifically implicating the free-embryo and larval stages as critical imprinting periods. Our study provides evidence for olfactory imprinting in a non-salmonid fish species, and supports further consideration of conservation tactics such as stream-side rearing facilities that are designed to encourage olfactory imprinting to targeted streams during early life stages. Continued research on lake sturgeon can contribute to a model of olfactory imprinting that is more generalizable across diverse fish species and will inform conservation actions for one of the world’s most imperiled fish taxonomic groups.

## Teaser

Natural odors guide reproductive migrations in many fish species, and odors experienced during early life are suspected to influence stream selection decisions by spawning adults, including lake sturgeon. We measured behavioral responses to artificial odorants in juvenile lake sturgeon and found evidence for olfactory imprinting during two early life stages.

## Introduction

Many fish species migrate from feeding grounds to spawning locations (Lucas and Baras, 2001). The timing and destination of spawning migrations have implications on the rate of early development and offspring survival (Reznick *et al.,* 2006; Forsythe *et al.,* 2012), and therefore are a mechanism by which adults provide indirect benefits to offspring (Leggett, 1977; Jørgensen *et al.,* 2008). Survival benefits conferred to offspring based on optimal spawning choices by parents select for repeated spawning at the optimal location, or spawning site fidelity, which can lead to localized adaptions and genetically distinct populations at different spawning sites (Leggett, 1977). In contrast, interbreeding caused by adults straying into other spawning sites may lead to outbreeding depression, which is the reduction in fitness because of the breakdown of coadapted genotypes that are adapted to species and (largely natal) environments (Edmands, 2007). Understanding the mechanisms guiding natal site homing is important for the management of migratory species, and studies have shown that straying from natal sites may occur more often by stocked individuals (Quinn, 1993).

Many fish species are hypothesized to use olfactory cues to home to natal habitat for spawning (Bett and Hinch, 2016). Natal homing via olfactory cues is well-studied in Pacific salmon (*Oncorhynchus* sp), which imprint to stream-specific odors during early life stages, especially the period of parr-smolt transformation, and then follow these odors at sexual maturity when navigating to spawning sites (Dittman and Quinn, 1996). In salmon and other species, research on olfactory imprinting informs conservation efforts because, for example, the findings support development of artificial propagation programs that encourage homing to targeted locations (Dittman *et al.,* 2015). Despite extensive research on olfactory imprinting in Pacific salmon and relevance of olfactory imprinting to management of various species, evidence for olfactory imprinting by non-salmonid species is largely indirect, such as observed homing to spawning sites or development of olfactory structures during suspected imprinting periods (Horrall, 1981; Cathcart, 2021).

The established model of olfactory imprinting in Pacific salmon is unlikely representative of many fishes (Bett and Hinch, 2016). Most research on olfactory imprinting to the odor of natal habitats in Pacific salmon has focused on coho salmon (*O. kisutch*; Bett and Hinch, 2016), which spend about a year in home streams after hatching, and while undergoing the metamorphosis-like transition of smolting (Bishop *et al.,* 2006; Björnsson *et al.,* 2012), move to the ocean to feed for about 1.5 years before returning to natal streams to spawn and die. However, many fish, including some species of Pacific salmon, differ from coho salmon in life history traits potentially related to olfactory imprinting, such as duration of pre-smolting occupancy of freshwater habitats. Species such as pink salmon (*O. gorbuscha*) and walleye (*Sander vitreus*) leave their natal habitats soon after hatching and would need to imprint much earlier than coho salmon (Horrall, 1981; Bett *et al.,* 2016). Likewise, most fish develop directly from embryos to juveniles to adults rather than undergoing any type of metamorphosis associated with major changes in levels of thyroid hormones (Rousseau and Dufour, 2012) that mediate imprinting in Pacific salmon (Dittman and Quinn, 1996; Lema and Nevitt, 2004). Examples of other life history traits likely relevant to the role of olfactory imprinting in natal homing, and that differ among species include, the habitats in which spawning occurs (e.g. lake vs streams), the age at which adults return to spawn, and whether the species is semelparous (dies after single spawning season) or iteroparous (spawns repeatedly across years). Research is needed to test whether and how olfactory imprinting might guide natal homing in fishes with life histories different from Pacific salmon (Bett and Hinch, 2016).

Lake sturgeon (*Acipenser fulvescens*) have a life cycle notably different from Pacific salmon (Peterson *et al.,* 2007), but are nonetheless hypothesized to home to natal streams to spawn based on a similar process of olfactory imprinting (Holtgren *et al.,* 2007). Age-0 lake sturgeon hatch 8–14 days post-fertilization (dpf), begin exogenous feeding 13–19 days after hatching, and move from the river into lakes after approximately four months (Holtgren and Auer, 2004; Benson *et al.,* 2005). Males feed and grow in lakes for 12–20 years and females 14–33 years before becoming sexually mature (Bruch and Binkowski, 2002; Thiem *et al.,* 2013; Dammerman *et al.,* 2019). Lake sturgeon movements and habitat occupation prior to sexual maturity or outside of the reproductive season vary among individuals and populations, and include year-around residence in the river, residence in a lake near the natal river and migrations across large portions of a lake (Kessel *et al.,* 2018; Scribner *et al.,* 2022). Once mature, males migrate into streams to spawn every 1–3 years and females every 4–9 years (Peterson *et al.,* 2007; Forsythe *et al.,* 2012). Like Pacific Salmon, lake sturgeon show spawning site fidelity, inferred based on high levels of genetic differentiation among populations (DeHaan *et al.,* 2006; Welsh *et al.,* 2008; Homola *et al.,* 2012; Donofrio *et al.,* 2018; Scribner *et al.,* 2022). Observations of spawning site fidelity in lake sturgeon and high straying rates in shortnose sturgeon (*A. brevirostrum*) reared in water from sources other than the river in which they were stocked (Smith *et al.,* 2002) have led to the hypothesis that olfactory imprinting guides natal homing in lake sturgeon (Holtgren *et al.,* 2007).

A better understanding of olfactory imprinting is needed to support efforts to restore self-sustaining and genetically diverse populations of lake sturgeon. Once among the most abundant fishes in the Laurentian Great Lakes, Mississippi River and Hudson Bay drainages (Scott and Crossman, 1973; Haxton *et al.,* 2014), lake sturgeon populations in most areas are now reduced to less than 1% of their historical numbers (Hay-Chmielewski and Whelan, 1997), and are considered extirpated, endangered, threatened, or of special concern in much of their range (Bruch *et al.,* 2016). Together with habitat improvements and fishery restrictions (Welsh, 2004), current efforts to restore lake sturgeon rely heavily on artificial propagation (Bruch *et al.,* 2016). In particular, lake sturgeon hatchery programs increasingly employ stream-side rearing facilities in an effort to imprint young sturgeon to targeted streams and to encourage subsequent homing by spawning adults (Holtgren *et al.,* 2007). Although tactics intended to encourage natal homing via imprinted stream odors are currently in use, whether and when age-0 lake sturgeon imprint to odors remains unknown.

In this study, we examined one essential facet of the hypothesis that olfactory imprinting guides natal homing in lake sturgeon: specifically, the ability to form olfactory memory (i.e. store and later retrieve olfactory information; Zlotnik and Vansintjan, 2019) and during early life stages (Hino *et al.,* 2009). Age-0 lake sturgeon leave their natal stream early in life (~ 4 months; Holtgren and Auer, 2004; Benson *et al.,* 2005) and undergo rapid forebrain development in key olfactory information centers during the transition from free-embryos to exogenously feeding larvae (Zhang and Dang, 2014). Studies in Russian sturgeon (*Acipenser gueldenstaedti*) have also provided evidence for olfactory imprinting during early development, finding elevated thyroid hormone levels prior to exogenous feeding (Boiko *et al.,* 2004) and demonstrating learned responses to morpholine in larval fish with elevated thyroid hormones (Boiko and Grigor'yan, 2002). Therefore, we predicted that imprinting—defined here as learning that occurs during a developmentally sensitive period and results in responses that persist outside of that period (Immelmann, 1975) in lake sturgeon occurs during the free-embryo (12–18 dpf) and larval stages (19–49 dpf). Although our overarching hypothesis pertains to odor-mediated homing by adult lake sturgeon, we tested behavioral responses of juveniles to odorants experienced during early life because raising individuals to adulthood (10–30 years) was not feasible. Research on Pacific salmon indicates young-of-year individuals display attraction to their natal river water (Bodznick, 1978), and this response has been a useful proxy for responses of adults to imprinted odors (Dittman *et al.,* 2015). We exposed age-0 lake sturgeon to artificial odorants during specific developmental stages and later tested their behavioral responses to the odorants to determine whether they were recognized during later life stages. Our experiments provided rare evidence for olfactory imprinting in a non-salmonid, identified periods during which imprinting likely occurs in lake sturgeon, and will inform restoration efforts that increasingly rely on hatchery stocking to rebuild or reintroduce populations in specific streams.

## Methods

### Experimental animals

Lake sturgeon used in experiments were reared from eggs fertilized at the Black Lake Sturgeon Rearing Facility in Onaway, MI, USA, which operates as a flow-through streamside rearing facility (SRF) using water supplied directly from the Upper Black River at ambient temperature (ranging from 8°C to 28°C over the course of the experiment). Eggs and sperm were sampled from spawning lake sturgeon in the Upper Black River on May 4, 2021. Eggs were fertilized within 8 hrs following standardized hatchery procedures (Crossman *et al.,* 2011; Bauman *et al.,* 2015). Offspring from one male and one female were used in the experiments. The use of full siblings was expected to reduce variation due to additive genetic effects (Dammerman *et al.,* 2015, 2020). Experimental animals were used with approval from the Michigan State University Animal Use and Care Committee (Animal Use Form # PROTO202000023/AMEND202100062).

Fish were raised in 68 L tanks in a flow-through system with 50 micron filtered stream water from the Black River. Within the 68 L tanks, fertilized eggs were held in McDonald hatching jars (Pentair, Apopka, FL) (held at a density of less than 200 eggs). Hatched fish were held in 3 L aquaria with bio ball filters (CBB1-S; Pentair, Apopka, FL) to simulate natural stream substrate (held at a density of less than 100 individuals), and exogenous feeding fish in 3 L aquaria without bio ball filters (held at a density that ranged from 25 to 50 individuals). Fish were moved to the larger 68 L tanks for the juvenile stage (held at a density of less than 50 individuals). While tank turnover rates varied by tank type, on average tanks experienced 10 turnovers per hour, and inflow rates were increased during periods of high temperatures during the summer months to reach closer to 40 turnovers per hour. Throughout our experiments, fish were fed a diet of brine shrimp (*Artemia* spp.) from 19 to 49 dpf and bloodworms (Diptera: Chironomidae) after 49 dpf.

### Exposure to experimental imprinting odorants

**Figure 1:**
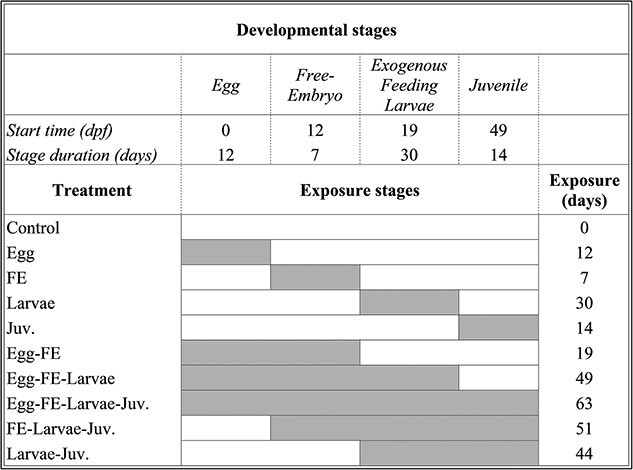
Timing and duration of the four developmental stages (Egg, Free-Embryo [FE], Exogenous Feeding Larvae [Larvae], and Juvenile [Juv.]) and ten experimental odorant exposure treatments. Start time for each developmental stage refers to dpf and stage duration refers to the total length (days) of each developmental stage. Exposure stages associated with each treatment are indicated in white (developmental stages without odorant exposure) and grey (developmental stages with odorant exposure). Exposure length was measured in days.

Fish were exposed to two experimental odorants that were continually mixed in 50 micron filtered river in the hatchery, phenethyl alcohol (PEA) at 1.04 x 10^−7^ M and morpholine at 9.9 x 10^−11^ M concentrations. Odorants were purchased from Sigma-Aldrich Co., Saint Louis, MO, USA at ≥99% purity. Odorants and concentrations were selected based upon olfactory imprinting studies in Pacific salmon (Bett and Hinch, 2016). PEA and morpholine are potent odorants for fish (Scholz *et al.,* 1976), allowing control of the exact concentrations and periods during which odorants were experienced without potentially confounding effects of background odorants. Odorant exposure occurred during four early developmental stages: (1) fertilized egg (0 dpf), (2) hatched free-embryos (12 dpf, ~ 9 mm total length), (3) exogenously feeding larvae (19 dpf, ~ 20 mm total length)—when individuals began feeding on brine shrimp (*Artemia* spp.), and (4) juveniles (49 dpf, ~ 45 mm total length)—when individuals began feeding on blood worms (Diptera: Chironomidae) (Fig. 1). Lake sturgeon exposure to odorants was organized into ten unique combinations of developmental stage treatment to determine whether olfactory imprinting occurred during a specific stage or combination of stages. Individuals in each treatment were raised in three replicate tanks, but some replicate tanks were lost due to mortality. Overall, mortality was low (less than 5% of fish) during this study, with small increases in fish mortality during high temperature events, which were unrelated to the timing of odor application. Individuals and replicates in one (control) treatment were never exposed to experimental odorants. Individuals and replicates in another treatment were exposed during all stages. Individuals and replicates in four treatments were exposed during a single developmental stage, and individuals and replicates in four treatments were exposed during two to three consecutive developmental stages including either the egg, free embryo (FE), exogenous feeding larvae, or juvenile (juv.) stage.

The developmental stages selected represent four distinct periods of development, behavior and location/habitats lake sturgeon occupy in natural stream environments when olfactory imprinting may occur. The beginning and end of each stage were determined by critical thermal units and physiology (Eckes *et al.,* 2015). The egg stage begins immediately with fertilization in the water column after which eggs adhere to the stream substrate. The FE stage begins at hatch and is the period when lake sturgeon burrow into the substrate to avoid predators and consume their yolk-sac (Kempinger, 1988; Detlaff *et al.,* 1993). The exogenous feeding larvae stage represents the period when fish have depleted their yolk-sac, begun feeding from the external environment and emerged from the gravel to drift downstream in river currents (Auer and Baker, 2002). Individual larvae typically drift for one to two days and the juvenile stage for our experiment occurred thirty days after larval drift, when lake sturgeon forage for food in juvenile habitat of their natal river system.

Odorant stock solutions were mixed daily in 3 L of hatchery water to create our odor mixture; a 10 ml stock solution was used for PEA and a 1 ml stock solution was used for morpholine. We calculated the concentrations for the stock solutions based on the final volume of the odor mixture and this mixture was applied to our system water to establish the proper concentration of each odorant in our experimental tanks. A peristaltic pump (model: BT100S, Golander, Duluth, GA) was used to pump the odor mixture into an 88 L head tank supplying water to tanks receiving the experimental odorants. Rhodamine dye (Sigma-Aldrich Co., Saint-Louis, MO, USA) was pumped into the head tank to reach a concentration of 100 ppb and measured using a hand-held DataBank datalogger and Cyclops-7 Optical Rhodamine Dye Tracer (Turner Designs, Sunnyvale, CA) to visually validate mixing of odorants in the head tank and to validate the even distribution of odorants supplied to all tanks. We recorded the time of odorant mixture replacement and the volume of odorant mixture remaining each day to track daily odorant concentrations across all tanks. Using the volume and duration of odorant mixture pumped each day, we calculated an average concentration of 9.71 ± 0.22 x 10^−8^ M (mean ± standard error [SE]) for PEA and 9.24 ± 0.21 x 10^−11^ M (mean ± SE) for morpholine over the duration of the experiment.

### Behavior experiments

Juvenile lake sturgeon swimming and activity behaviors were observed in response to PEA and morpholine as a test of olfactory memory of the artificial natal odorants. Twenty individuals were observed from each treatment, using an equal number of individuals for each replicate. Trials took place in a cylindrical tank with a 1552 cm^2^ (44.4 cm diameter) base filled with 3 L (or a depth of 1.9 cm) of groundwater from the facility (Supplementary Fig. S1). Four identical arenas were used to measure behaviors based on multiple estimated movement parameters, allowing for multiple trials to be run concurrently. Prior to experiments we measured behavioral responses to food odors (bloodworms) and spring water (negative control) to verify our behavioral assay could detect changes in behavior after exposure to a specific odorant and not simply any substance. For each trial, one individual fish was removed from its housing tank and a photo was taken for body length measurements. Fish were then acclimated to the enclosure for seven and a half minutes which, based on preliminary observations, was the time when total activity reduced to normal levels for most individuals. Videos were recorded for five minutes after acclimation to measure pre-odor behaviors. Odorant solutions were created to reach the desired concentrations of 1.04 x 10^−7^ M for PEA and 9.90 x 10^−11^ M for morpholine in the behavioral arena. A volume of 100 ml of odorant stock solution was added using two 50 ml syringes; observers were instructed to add the odors to the water using a zig-zag pattern evenly across the surface in the behavioral arena over a five second period. Applying the odors across the surface using two syringes resulted in even application and mixing of the odorant throughout the arena, as confirmed using dye tests. One minute after the initial addition of odorants, another five-minute video was recorded to measure behaviors post-odor application. Fish were then removed from the enclosures, and enclosures were thoroughly rinsed with groundwater before the next trial began. To prevent outside stimuli from affecting fish behaviors, fish were observed in the evening outside of working hatchery hours and in the dark under red lighting. Length was measured for each fish with the ImageJ software (National Institutes of Health, Bethesda, MD, U.S.A.; http://rsbweb.nih.gov/ij/). Videos were analyzed using Loligo v.4.0 tracking software (Loligo Systems, Viborg, Denmark; https://www.loligosystems.com/software), which recorded average velocity (cm/s), average acceleration (cm/s^2^), average deacceleration (cm/s^2^), time active (s), time active (%), time inactive (s), time inactive (%) and total distance traveled (cm).

### Statistical analyses

All analyses were conducted using R version 4.1.2 (R Core Team, 2021). To identify relationships between recorded behavioral movement metrics and to select an informative metric to use in our analysis, we calculated correlations between response variables using the *corrplot* package (v0.92; Wei and Simko, 2021). Using the absolute value of Pearson correlation coefficients, we found strong pairwise correlations (|r| ≥ 0.89) between average velocity, average acceleration, average deacceleration and total distance traveled variables (Supplementary Fig. S2). We also found strong correlations (|r| ≥ 0.93) between time active and time inactive measures. There was a moderate correlation (|r| > 0.62) between distance traveled and all time active and inactive measures. Pairwise correlations between all potential response variables were non-zero (p < 0.001). Due to its correlation with the other response variables, total distance moved during the five-minute observation period was selected as the single representative behavior to be used in our statistical analyses of odorant response.

Prior to statistical analysis, we reviewed data for visual and statistical outliers in both pre-odor and post-odor distance traveled. Given our data were generated using a tracking software to analyze video recordings, we inspected the data to ensure no outliers were included that may have resulted from a tracking anomaly. Results from each trial were inspected visually to identify abnormal and extreme values of distance traveled by an individual. A Grubbs Test, which identified extreme outliers from the assumed normal distribution of the distance traveled measure in each treatment group, was conducted using the *outliers* package (v0.14; Komsta, 2011) to verify our visual observations. Two out of one hundred and ninety-six trials were removed as outliers based upon visual inspection and evaluation using the Grubbs Test. Four additional trials were removed because of tracking related issues (e.g. lighting variation) or incomplete video recordings.

We modeled post-odor distance traveled under a normal distribution using robust linear regression as a function of predictor variables measured throughout the experiment. Normality of residuals and homoscedasticity were assessed following model selection for a traditional linear regression. The model did not meet the assumptions. Specifically, we observed multiple highly influential (high leverage) observations in our model based on the residual quantile-quantile and residuals vs. leverage plots (Chatterjee and Hadi, 1986). Based on these findings, we performed robust linear regression models using an M estimator, which down-weighs highly influential observations without removing observations from the analysis (Filzmoser and Nordhausen, 2021). Models were compared to select fixed effects to include in full model predictions and inference. To account for individual variation in swimming behaviors and activity, pre-odor distance traveled was included as a predictor variable in all but the null model. The fixed effects included individual length, pre-odor distance traveled, treatment group and their pairwise interactive effects. Models were compared using Akaike Information Criterion—small sample size correction (AICc) with the *AICcmodavg* package (v2.3–1; Mazerolle, 2020). All models within two AICc of the top model were considered (Tredennick *et al.,* 2021).

Robust linear mixed models were run based on results from the fixed effects model selection using the *robustlmm* package (Koller, 2016). Models also included arena as a random intercept, tank as a random intercept, or both arena and tank as crossed random effects to account for non-independence of individuals based on these factors. However, the estimated effect of tank was 0 so only the random effect of arena was included to prevent overfitting. Random effects were included for model interpretation but not selection, as we were not interested in making inferences on the random effects (Gomes, 2022) and there is no accurate method for robust linear mixed model comparisons (Koller, 2016). Figures were produced for the focal model using the *ggplot2* (Wickham, 2016) and *cowplot* (v1.1.1; Wilke, 2020) packages. Predictions based on robust linear models were made using the *predict.rlm* function from the *MASS* package and included only fixed effects because the *predict.rlm* function cannot generate confidence intervals for mixed models (Venables and Ripley, 2002).

## Results

**Figure 2:**
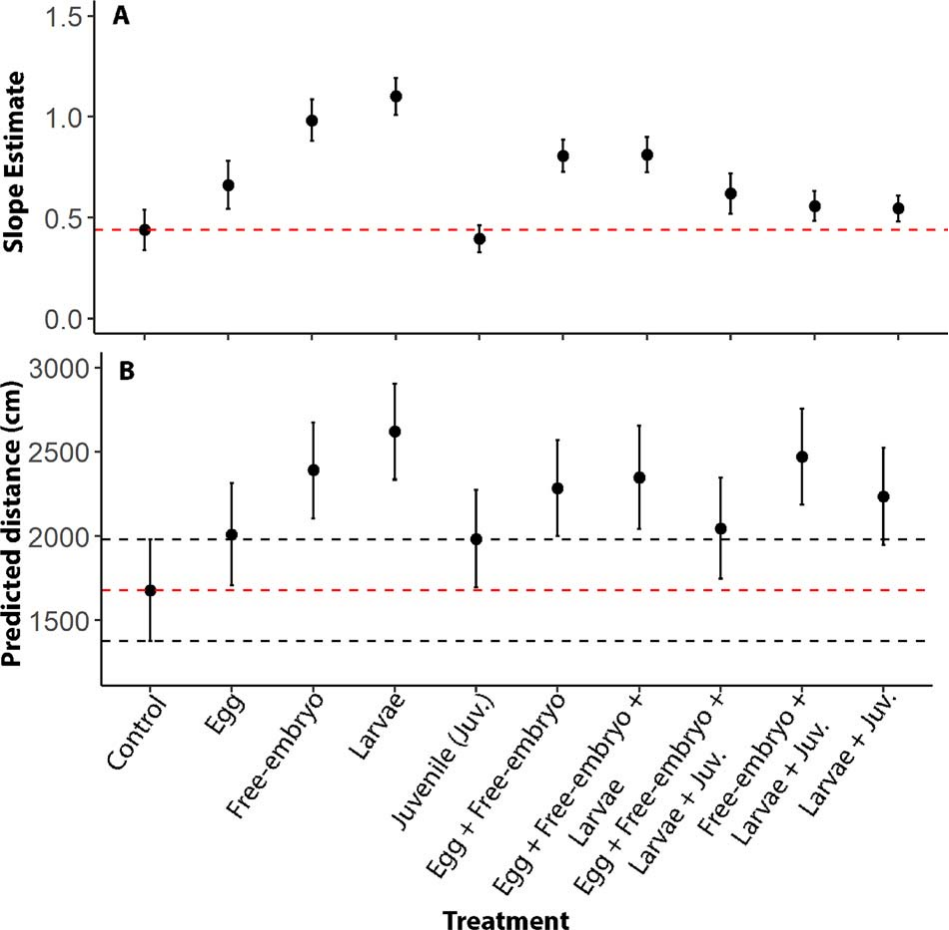
Slope estimates for the relationship between pre-odor distance traveled (A) and predicted post-odor distance traveled at the mean of 2635 cm after 1000 simulations (B) based on the robust linear model relating post-odor distance to treatment, pre-odor distance and the interaction between treatment and pre-odor distance, which was identified as the top model based upon AICc values (see Table 1). Error bars represent one SE of the slope estimates (A) and 95% confidence intervals of predicted responses (B). Red dashed horizontal lines were included for comparison between the control and other treatment groups and represent the estimated slope value and predicted post-odor distance for the control. Black dashed horizontal lines represent the value of the lower and upper 95% confidence intervals of the post-odor distance prediction for the control group. Numbers above the estimates represent sample sizes for each treatment.

Lake sturgeon reared in water containing PEA (1.04 x 10^−7^ M) and morpholine (9.90 x 10^−11^ M) during early life stages traveled a greater distance after exposure to the odorants in behavioral experiments compared to naïve (control) individuals. AICc values indicated the best-fit model included pre-odor distance traveled, treatment and their interaction (Table 1). Pre- and post-odor distance traveled were positively correlated across all treatments, though this relationship varied by treatment (Fig. 2A; Table 2). All groups exposed to PEA and morpholine, except for the group exposed only during the juvenile stage, had a larger increase in post-odor distance traveled with a unit increase in pre-odor distance traveled (slope) compared with fish never exposed to PEA or morpholine prior to behavioral testing. Predicted distances traveled and 95% confidence intervals based upon our best-fit model, and a constant pre-odor distance (2635 ± 163 cm; mean ± SE) indicated the exogenous feeding and free-embryo stages were most important (Fig. 2B); fish exposed during the exogenous feeding stage only had the highest predicted post-odor distance traveled response at the mean pre-odor distance traveled (56% larger than the control), followed by fish exposed during the free-embryo stage only (42% larger than control). Predicted post-odor distance traveled responses at the mean pre-odor distance and slope estimates were not larger for fish exposed during consecutive stages when compared to fish exposed during the free-embryo or exogenous feeding stages only (Fig. 2B).

**Table 1:** Comparison of AICc scores of robust linear regression models for post-odor distance traveled responses associated with different fixed-effects

Model	ΔAICc
Treatment + pre-odor distance + treatment*pre-odor distance	0
Treatment + pre-odor distance + length + treatment * pre-odor distance	2.71
Pre-odor distance	3.53
Length + pre-odor distance + length * pre-odor distance	5.42
Length + pre-odor distance	5.48
Treatment + pre-odor distance	13.34
Treatment + pre-odor distance + length	15.57
Treatment + pre-odor distance + length + length * pre-odor distance	16.94
Treatment + pre-odor distance + length + treatment * pre-odor distance + length * pre-odor distance + treatment * length	18.26
Treatment + pre-odor distance + length + treatment * length	23.4
Intercept only	184.45

AICc scores are calculated in reference to the model with the lowest AICc. Fixed effects included treatment, pre-odor distance, total length of the individual and pairwise interactions between the independent variables.

## Discussion

As discussed below, our study does did not directly investigate adult homing via olfactory imprinting during earlier life stages, as we only studied behavioral responses of juveniles. Nevertheless, the results support the hypothesis that lake sturgeon imprint to odors experienced during early life stages. Age-0 lake sturgeon reared in water activated with PEA and morpholine for as little as 7 days responded to the odorant mixture in behavioral (movement distance) assays over 50 days after the initial odorant exposure. The critical imprinting window included the free-embryo and the exogenous feeding (larval) stages, as exposure to the odorants during these periods resulted in elevated responses during subsequent behavioral testing. In contrast, exposure during egg or juvenile stages yielded responses no different than control treatments in which fish were naïve to the odorants prior to behavioral testing. Taken together, the results provide the first direct evidence for olfactory imprinting in lake sturgeon, and implicate the free-embryo and larval stages as developmental periods important for imprinting.

Experimental evidence for embryonic and larval imprinting aligns with established aspects of lake sturgeon ecology and developmental biology (Zhang and Dang, 2014). Age-0 lake sturgeon remain close to stream spawning and hatching locations through the onset of exogenous feeding (13–19 days after hatch). Subsequently, downstream dispersal begins and juveniles leave streams within ~ 4 months (Holtgren and Auer, 2004; Benson *et al.,* 2005; Peterson *et al.,* 2007). Unlike species of Pacific salmon that remain in their natal stream for over a year and can imprint after age 1 (Dittman *et al.,* 1996), lake sturgeon must imprint to stream odors during early life stages if these odors guide natal homing by sexual mature adults some 15–20+ years later (Peterson *et al.,* 2007). Notably, previous morphological studies on age-0 lake sturgeon indicate rapid development of the olfactory bulb during the transition from free-embryos to exogenously feeding larvae (Zhang and Dang, 2014). In Russian sturgeon, physiological and behavior studies provide evidence for olfactory imprinting and elevated thyroid hormones during larval stages (Boiko and Grigor'yan, 2002; Boiko *et al.,* 2004). Our study provides behavioral evidence for olfactory imprinting during the free-embryo and larval stages in lake sturgeon, though it does not exclude the possibility of additional windows of imprinting later during the juvenile stage because we only attempted to imprint fish up to approximately two months post-fertilization.

Whether the free-embryo and larval stages represent one continuous or two distinct imprinting periods in lake sturgeon remains unclear. Juvenile lake sturgeon responded to the artificial odorants after being reared in them during the free-embryo and larval stages combined or either stage individually. In Pacific and Atlantic salmon (*Salmo salar*), olfactory imprinting occurs primarily during the parr-smolt transformation, which is an important period of behavioral, endocrine and physiological changes (Hasler and Scholz, 1983; Morin *et al.,* 1989). Recent studies have also provided evidence for olfactory imprinting at earlier stages (Dittman *et al.,* 2015; Bett *et al.,* 2016; Armstrong *et al.,* 2022), indicating olfactory imprinting may occur sequentially at multiple developmental stages and may guide natal homing not only to a specific river but to a specific natal site (Quinn *et al.,* 2006). Lake sturgeon remain near the spawning site burrowed in the substrate at the free-embryo stage (Kempinger, 1988) but leave the substrate and drift downstream during the exogenous feeding larvae stage (Auer and Baker, 2002), and could possibly imprint to slightly different odors during each stage. Although fine-scale natal homing has not been shown in sturgeon, observed use of separate spawning sites by distinct groups of lake sturgeon within a single river (Forsythe *et al.,* 2012) could be driven, in part, by imprinting during the free-embryo stage, whereas imprinting during the larval stage could guide coarse-scale homing to a river. Alternatively, the free-embryo and larval stages could represent a single window during which imprinting occurs, with exposure to an odor during only part of the window sufficient for imprinting. Unlike Pacific salmon (Havey *et al.,* 2017), lake sturgeon did not show stronger responses after exposure during two developmental stages versus one. Additional research and an experimental design that can directly compare responses to odors experienced during each stage is needed to determine whether the free-embryo and larval stages represent two distinct imprinting windows.

**Table 2:** Parameter estimates and SEs for the robust linear regression model of post-odor distance traveled based on treatment group (e.g. Free-Embryo [FE] or Juvenile [juv.]), pre-odor distance traveled, and the interaction between treatment and pre-odor distance traveled

Parameter	Estimate	SE
*Fixed-only*		
Intercept	521.229	283.05
Egg	−254.647	424.32
FE	−720.232	427.00
Larvae	−802.955	418.29
Juv.	423.184	402.24
Egg-FE	−360.368	404.10
Egg-FE-larvae	−311.486	394.62
Egg-FE-larvae-juv.	−108.347	430.44
FE-larvae-juv.	480.305	393.42
Larvae-juv.	276.980	393.30
Pre-odor dist.	0.440	0.10
Egg * pre-odor dist.	0.223	0.16
FE * pre-odor dist.	0.543	0.14
Larvae * pre-odor dist.	0.662	0.14
Juv. * pre-odor dist.	−0.045	0.12
Egg-FE * pre-odor dist.	0.366	0.13
Egg-FE-larvae * pre-odor dist.	0.372	0.13
Egg-FE-larvae-juv. * pre-odor dist.	0.180	0.14
FE-larvae-juv. * pre-odor dist.	0.118	0.12
Larvae-juv. * pre-odor dist.	0.106	0.12
*Fixed + Arena as a random intercept*		
Intercept	858.464	329.28
Egg	−417.863	435.75
FE	−877.482	438.37
Larvae	−1080.007	433.89
Juv.	281.279	418.70
Egg-FE	−569.650	417.14
Egg-FE-larvae	−498.138	405.07
Egg-FE-larvae-juv.	−423.665	441.74
FE-larvae-juv.	312.664	403.80
Larvae-juv.	144.816	403.88
Pre-odor dist.	0.246	0.10
Egg * pre-odor dist.	0.339	0.16
FE * pre-odor dist.	0.683	0.15
Larvae * pre-odor dist.	0.807	0.14
Juv. * pre-odor dist.	0.102	0.13
Egg-FE * pre-odor dist.	0.518	0.13
Egg-FE-larvae * pre-odor dist.	0.515	0.14
Egg-FE-larvae-juv. * pre-odor dist.	0.388	0.14
FE-larvae-juv. * pre-odor dist.	0.264	0.13
Larvae-juv. * pre-odor dist.	0.254	0.12

Estimates on the left are from the fixed-effects only models and estimates on the right are from a robust linear mixed model with the arena used for observations as a random intercept.

Challenges associated with the life history of lake sturgeon imposed constraints on our study that obfuscate the ecological implications of our results. The 10- to 30-year pre-reproductive stage of lake sturgeon (Peterson *et al.,* 2007) precluded use of the approach often taken with Pacific salmon, in which adults are tested for attraction to odorants they experienced during early development (Bett *et al.,* 2016). Observing juvenile responses to imprinted odorants proved to be a useful alternative that provided evidence for early life olfactory imprinting, but the ecological function of juvenile responses and the link to homing behavior of adults remain unknown. Responses of age-0 fish to imprinted odors in other species are related to kin recognition (zebrafish; Gerlach *et al.,* 2008) and larval settlement (coral reef fishes; Gerlach *et al.,* 2007). In the case of Pacific salmon, natal water preference by emergent fry has been documented, though it is unclear what the ecological function of this response is and how this behavior relates to the natal homing behaviors in adult salmon (Bodznick, 1978; Dittman *et al.,* 2015). Our behavioral assays were not designed to evaluate the ecological function of juvenile responses to imprinted odorants in lake sturgeon; however, the observed increase in total distance traveled after exposure to imprinted odorants could conceivably relate to dispersal of juveniles away from their natal habitat. Interestingly, a previous study found that lake sturgeon stocked in Black River were more likely to be recaptured downstream of the release site if they were reared in, and therefore potentially imprinted upon, Black River water versus water from an offsite hatchery (Crossman *et al.,* 2011). Although the observed differences in recapture rates were likely due, at least in part, to higher mortality of fish reared offsite (Crossman *et al.,* 2011), the results could also be explained by a greater propensity of Black River-imprinted fish to disperse from natal habitat downstream to novel feeding habitats. Future research on olfactory imprinting in lake sturgeon could continue to leverage juveniles as a proxy of homing adults while also elucidating the ecological function of juvenile responses to imprinted odorants.

The results of our study inform efforts to manage lake sturgeon, especially guidance pertaining to use of stream-side rearing facilities (Holtgren *et al.,* 2007). First, our experiments provided behavioral evidence that lake sturgeon can imprint to odors experienced during early life stages, a critical prediction of the hypothesis that the genetic structure of lake sturgeon results from natal homing via imprinted stream odors (DeHaan *et al.,* 2006; Welsh *et al.,* 2008; Homola *et al.,* 2012; Donofrio *et al.,* 2018; Scribner *et al.,* 2022). Although stream-side rearing facilities also increase survival of age-0 lake sturgeon (Crossman *et al.,* 2011), a major objective of their use is to imprint lake sturgeon to targeted rivers and encourage adult homing to those rivers (Holtgren *et al.,* 2007). Second, evidence reported here and elsewhere (Boiko and Grigor'yan, 2002; Boiko *et al.,* 2004; Zhang and Dang, 2014) suggesting that olfactory imprinting occurs during the free-embryo and larval stages implies imprinting lake sturgeon to targeted rivers may be most effective when fish are brought into stream-side rearing facilities during early life stages. Notably, many stream-side rearing facilities raise lake sturgeon captured as drifting larvae in the target stream or from other higher-producing streams (Holtgren *et al.,* 2007). Our results suggest these fish may have already begun to imprint as free-embryos, which could affect the success of olfactory imprinting to other streams. Additional research is needed to determine the specific roles of imprinting during the free-embryo versus larval stages, especially for stream-side rearing facilities that rely on lake sturgeon caught as larvae from other rivers.

In conclusion, we provide behavioral evidence for embryonic and larval imprinting to odorants in a non-teleost with a life history markedly different from Pacific salmon. Importantly, we addressed only one prediction of the hypothesis that olfactory imprinting guides natal homing in lake sturgeon—the ability to respond to odorants experienced during specific life stages. However, numerous aspects of the sturgeon life cycle strain the model of natal homing via olfactory imprinting that has been developed for Pacific salmon (Dittman *et al.,* 2015). In particular, the delayed age-at-maturity raises questions about the stability of a stream’s signature odor and the persistence of lake sturgeon’s memory for it. Likewise, migratory patterns differ among populations of lake sturgeon and some migratory behaviors, such as moving downstream into lake outflows to spawn (Kessel *et al.,* 2018), are unlikely guided by olfactory cues in the same way as the migratory behaviors of Pacific salmon. Continued research on lake sturgeon is likely to inform conservation of one of the world’s most imperiled families of fish (Billard and Lecointre, 2000), and contribute to a model of olfactory imprinting that is more generalizable across diverse fish species.

## Funding

This work was supported by the Great Lakes Fishery Trust [project ID 1785].

## Author Contributions

J.G.K. collected and analyzed data, prepared figures and led manuscript writing. D.L.L assisted in data collection. T.J.B., E.A.B., T.G.Z., K.T.S. and W.L. procured funding. All authors helped with conceptualization of the study, methodology development and assisted in writing and editing of the manuscript.

## Data availability

The data underlying this article are available at the Dryad Digital Repository https://doi.org/10.5061/dryad.0p2ngf25t.

## Supplementary Material

Web_Material_coad045
