# Supplementary material for: Behavioral evidence of olfactory imprinting during embryonic and larval stages in lake sturgeon
Source: Conserv Physiol. 2023 Jul 3;11(1):coad045. doi: 10.1093/conphys/coad045 (PMC10317470; doi:10.1093/conphys/coad045)
Supplement: Web_Material_coad045 [file web_material_coad045.pdf]

Supplementary materials

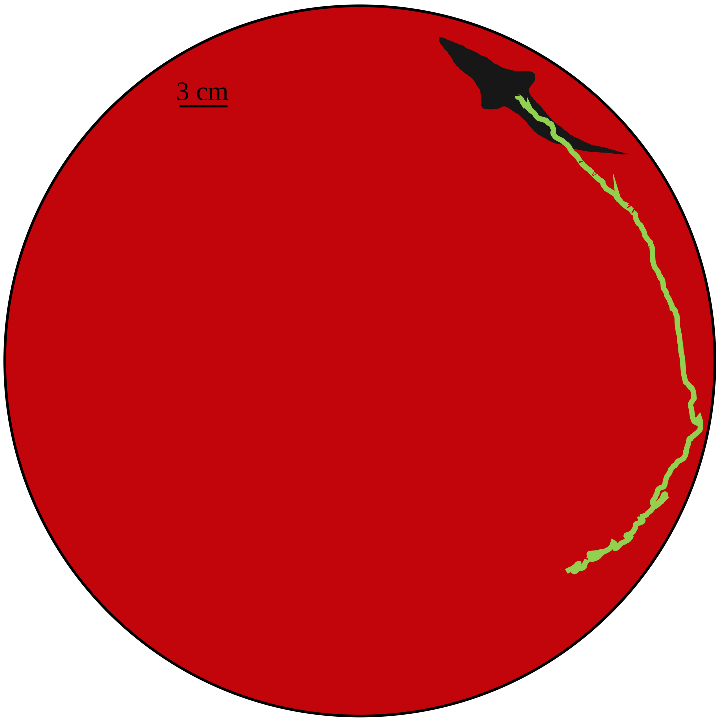

Figure S1. Example of a behavioral arena used for measuring responses of juvenile lake sturgeon to experimental odorants. Green tracking line represents fish tracking by the Loligo tracking software over a 10 second period.

	Avg. acceleration	Avg. deacceleration	Time active (%)	Time active (s)	Time inactive (s)	Time inactive (%)	Distance Traveled
Avg. velocity	**0.98**	**-0.98**	**0.36**	**0.35**	**-0.34**	**-0.36**	**0.89**
Avg. acceleration		**-1.00**	**0.27**	**0.26**	**-0.27**	**-0.27**	**0.84**
Avg. deacceleration			**-0.27**	**-0.26**	**0.27**	**0.27**	**-0.84**
Time active (%)				**0.99**	**-0.97**	**-1.00**	**0.66**
Time active (s)					**-0.93**	**-0.99**	**0.66**
Time inactive (s)						**0.97**	**-0.63**
Time inactive (%)							**-0.66**

Figure S2. Matrix showing pairwise correlations between all measured post-odor behavioral responses of juvenile lake sturgeon from the Loligo tracking software. All correlations were non-zero ($p<0.001$).
